# Multilocus Sequence Typing and Antimicrobial Susceptibility of *Listeria monocytogenes* Isolated from Foods Surveyed in Kosovo

**DOI:** 10.3390/microorganisms12122441

**Published:** 2024-11-27

**Authors:** Besart Jashari, Beatrix Stessl, Benjamin Félix, Armend Cana, Bledar Bisha, Dean Jankuloski, Katerina Blagoevska, Adeoye J. Kayode

**Affiliations:** 1 Food Microbiology, Food and Veterinary Laboratory, Food and Veterinary Agency of Kosovo, Lidhja e Pejës 241, 10000 Pristina, Kosovo; 2 Unit of Food Microbiology, Centre for Food Science and Public Veterinary Health, Clinical Department for Farm Animals and Food Systems Safety, University of Veterinary Medicine Vienna, Veterinärplatz 1, A-2110 Vienna, Austria; beatrix.stessl@vetmeduni.ac.at; 3 Laboratory for Food Safety, Salmonella and Listeria Unit, ANSES, European Union Reference Laboratory for *L. monocytogenes*, University of Paris-Est, 94700 Maisons-Alfort, France; benjamin.felix@anses.fr; 4 Microbiology Laboratory, University for Business and Technology-Higher Education Institution, Kalabria, 10000 Pristina, Kosovo; armend.cana@ubt-uni.net; 5 Department of Animal Science, College of Agriculture and Natural Resources, University of Wyoming, Laramie, WY 82071, USA; bbisha@uwyo.edu; 6 Food Institute, Faculty of Veterinary Medicine-Skopje, Ss. Cyril and Methodius University in Skopje, Lazar Pop-Trajkov 5-7, 1000 Skopje, North Macedonia; djankuloski@fvm.ukim.edu.mk (D.J.); katerinab@fvm.ukim.edu.mk (K.B.); 7 Department of Biochemistry, Genetics and Microbiology, University of Pretoria, Private Bag X20, Hatfield, Pretoria 0028, South Africa; johnkayus3749@gmail.com

**Keywords:** *L. monocytogenes*, sequence typing, multidrug-resistant, food chain, antimicrobial resistance, intrinsic resistance

## Abstract

In the absence of data on the reporting of *L. monocytogenes* resistance to antibiotics, we sought to determine which clonal complexes (CCs)/sequence types (STs) circulate in the food chain in Kosovo and to determine their antibiogram profiles to a panel of 18 antibiotics. From a total of 114 isolates, 21 different typical STs were identified by multilocus sequence typing (MLST). Each isolate derived from the food categories was subjected to tests to verify its susceptibility to the selected antibiotics according to the designed Sensititre GPN3F panel. Among the different STs that were identified, CC9-ST9 was more abundant in meat products (38.75%) while CC29-ST29 was more abundant (24.0%) in dairy products. Moreover, these isolates showed marked resistance against levofloxacin (22.8%), gentamicin and rifampicin (17.5%), quinupristin/dalfopristin (14.9%), erythromycin (11.4%), penicillin (7.89%), tetracycline (1.75%), and streptomycin (0.88%). A total of 27 multiple antibiotic resistance (MAR) phenotypes were observed amongst the isolates, which ranged from 3 to 12. The ARI of the food category including meat and meat products (MMP, 0.22) and fish meat products (FMP, 0.26) were >0.2, the permissible Krumperman threshold. The number of strains with MAR values >0.2 was 34, (29.8%). The identification of typical multidrug-resistant STs among *L. monocytogenes* isolates in Kosovo constitutes a potential threat to food safety and public health, which requires a continuous and expanded surveillance system to prevent the further spread of antimicrobial resistant (AMR) isolates.

## 1. Introduction

*Listeria monocytogenes (L. monocytogenes)* is a psychrotrophic pathogen known for its ability to not only survive but also grow at temperatures below 1 °C, as well as its characteristic tolerance to salt, making it persistent in food processing environments and capable of contaminating food products [1,2,3]. Listeriosis is a zoonotic/foodborne infection that is mainly acquired from the consumption of contaminated food products, which, in addition to economic losses with high treatment costs, is known for its high mortality rates (20–30%) in humans [4,5,6]. In the European Union (EU) in 2022, *L. monocytogenes* was considered the cause of the most serious human infection that mainly comes from contaminated food, with 2738 confirmed cases of the disease and 35 outbreaks of listeriosis of food origin. During this period in the EU, a mortality rate of 15.9% was reported from hospitalized cases [7].

*L. monocytogenes* is genetically organized into four phylogenetic lineages, with lineages I and II commonly associated with outbreaks resulting from the consumption of contaminated food [8,9]. To date, 13 serotypes and 4 molecular serogroups have been identified by serological and molecular methods [10,11], of which over 95% are the cause of invasive listeriosis in humans [8,12]. The advancement of molecular techniques has enabled the identification of clonal complexes or epidemiological clones of *L. monocytogenes*, which are now used to differentiate genetically similar isolates that are involved in both isolated and large-scale outbreaks [13,14,15]. Multilocus sequence typing (MLST) is a technique that assesses the allelic similarity of multiple housekeeping gene loci by direct DNA sequencing. This method allows for a comprehensive epidemiological investigation of *L. monocytogenes* and more accurate tracing of the sources of specific strains during outbreaks [15,16,17,18]. In a study conducted from 2016 to 2022 in Kosovo, 16 groups of CCs were identified using real-time PCR, including highly hypervirulence strains [19].

Recently, the use of compounds with bacteriostatic and bactericidal effects against *L. monocytogenes* has grown unchecked. The increasing trend of the unrestricted application of synthetic and semi-synthetic antimicrobials is contributing to the phenomenal increase in antimicrobial resistance [20,21,22]. In treating *L. monocytogenes*, the antibiotics that are considered the “gold standard” include aminopenicillins (ampicillin or amoxicillin), benzylpenicillin (penicillin G and gentamicin) used in combination with aminoglycosides, trimethoprim alone (or combined with sulfamethoxazole), erythromycin and tetracyclines [23,24,25]. For immunocompromised individuals and pregnant women, alternative therapies may include vancomycin, trimethoprim–sulfamethoxazole, erythromycin, carbapenems, or piperacillin–tazobactam, respectively, are given as alternative therapy [26,27]. Additionally, *L. monocytogenes* exhibits an intrinsic resistance to a wide spectrum of antibiotics, including fosfomycin, fusidic acid, cephalosporins, and various cephalosporins such as cefetam, cefotaxime, ceftriaxone, and cefuroxime [25,28].

To date, the widespread resistance of *L. monocytogenes* to a broad range of antibiotics has not been evident. However, the first multidrug-resistant (MDR) strain was identified in 1988. Since then, MDR strains against multiple antibiotics have been observed in cases of listeriosis outbreaks [29].

To the best of our knowledge, there are no reports on the antimicrobial resistance of *L. monocytogenes*, or isolates identified by molecular methods in the food chain in Kosovo. According to a report from the Kosovo National Institute of Public Health, only a small number of human listeriosis cases have been registered, reflecting the sporadic nature of listeriosis in the region. Kosovo is located in southeastern Europe, at the centre of the Balkan Peninsula, and has a favourable geographical position for the development of agriculture and livestock, presenting a promising economy for the future. Additionally, there is a lack of data on the use of antibiotics in animals, which plays a crucial role in the transmission of antibiotics through food to humans.

Studies on the antibiotic sensitivity of *L. monocytogenes* have been conducted in various countries within the region and beyond, utilizing phenotypic methods, such as disc diffusion and minimum inhibitor concentration (MIC) [20,30,31,32].

Therefore, this study is crucial as it is the first to investigate antimicrobial resistance in *L. monocytogenes* isolates from the food production chain in Kosovo, a region with no prior data on this public health concern. Given the central position of Kosovo in the Balkan Peninsula and its promising agricultural potential, understanding antimicrobial resistance patterns in foodborne pathogens is essential for safeguarding both public health and food supply. Our study not only identifies key epidemiological populations of *L. monocytogenes* but also reveals their antibiogram signatures to commonly used antibiotics in veterinary medicine. This baseline data are vital for establishing informed antibiotic stewardship and mitigating the potential risk of multidrug-resistant pathogens in Kosovo.

Our study aimed to identify the epidemiologic populations of Sequence types (STs) among 114 strains of *L. monocytogenes* isolated from the food chain examining their correlation with resistance against 18 antibiotics used in veterinary and human therapy as well as assessing the occurrence of multidrug-resistant *L. monocytogenes* isolates in the Kosovo region.

## 2. Materials and Methods

### 2.1. Sampling

In this study, 114 strains of *L. monocytogenes* isolated from food products in the food production chain in Kosovo, collected between 2016 and 2022. The samples include products of animal origin such as meat and meat products, milk and milk products, combined animal-based food products, fish, and environmental samples from food processing facilities. Food products of animal origin were collected from food business operators from meat and meat products (*n* = 80) with the subcategories of beef meat products (*n* = 69); pork meat products (*n* = 9); chicken meat products (*n* = 1); sheep meat products (*n* = 1); milk and dairy products (*n* = 25) with the following subcategories of dairy products (*n* = 21): fresh milk (*n* = 4) and fish meat products (*n* = 4); combined food products (*n* = 4) with the following subcategories: burgers with cheese (*n* = 2); chicken fillets with cheese (*n* = 2); and an environmental sample of a food-contact surface (*n* = 1). Expressed as a percentage, the representation of samples from food categories in this study was 70.2% isolates from meat and meat products; 21.9% milk and milk products; 3.51% fish products; 3.51% combined food products; and 0.88% were from sample workspaces. Samples were collected under aseptic conditions from business operators in Kosovo including factories, slaughterhouses, retail shops, cold rooms, and restaurants and were transported in an insulated cooling box to the microbiology laboratory for processing. The study was conducted at the University of Veterinary Medicine Vienna in Austria, the Institute of Food Safety, Food Technology and Veterinary Public Health, and the Food Microbiology Unit. All samples were processed within 6 h after collection.

### 2.2. Detection of L. monocytogenes Isolates

The method used in this study for detection and isolation was based on the EN ISO 11290–1 [33] International Organization for Standardization standard which is a two-stage enrichment procedure. For the initial selective enrichment, Demi–Fraser Broth (DFB, Liofilchem^®^ S.r.l., Roseto degli Abruzzi, TE, Italy) was used in relation to the sample of 25 g/225 mL supplemented with a acriflavine content of 0.0125 g/L and 0.01 g/L of nalidixic acid incubated at 30 °C for 24 ± 2 h. Then 0.1 mL of the broth culture from the primary enrichment was inoculated into 10 mL of *Listeria* Fraser Broth (LFB, Liofilchem^®^ S.r.l., Roseto degli Abruzzi, TE, Italy) supplemented with acriflavine 0.025 g/L and nalidixic acid 0.02 g/L and incubated at 37 °C for 24 h. *Listeria* agar Ottaviani and Agosti chromogenic nutrient medium (ALOA, Liofilchem^®^ S.r.l., Roseto degli Abruzzi, TE, Italy) and *Listeria* Oxford agar (LOA, Liofilchem^®^ S.r.l., Roseto degli Abruzzi, TE, Italy) were used as selective solid media, and the plates were incubated at 37 °C for 24–48 h. Presumptive strains were confirmed using biochemical tests, fermentation of rhamnose, and xylose sugars, haemolysis test and CAMP test [33]. Serological confirmation was performed using specific antisera (Denka Seiken, Tokyo, Japan). The preservation of the isolates was conducted in Microbank^®^ 2D vials (Prolab Diagnostics™, Richmond Hill, ON, Canada) at −80 °C.

### 2.3. DNA Extraction

Pure colonies were streaked on RAPID’L. mono agar (Bio-Rad, Hercules, CA, USA) for 24 h at 37 °C, then one colony was re-seeded on Tryptic Soya Agar (TSA, Merck, Darmstadt, Germany) and incubated for 24 h at 37 °C. For the extraction of DNA from TSA plates, 3–4 colonies were taken and suspended in 2 mL tubes containing 100 μL of 0.01 M Tris HCI pH 7 (Thermo Scientific, Rockville, MD, USA). Four hundred (400) microliters of Chelex 100 Resin solution were then added (Bio-Rad, Hercules, CA, USA). The contents were vortexed and then boiled at a temperature of 100 °C for 10 min. After boiling, the tubes were centrifuged for 5 min at 15,000 rcf. Using a pipette, 100 ul of supernatant was taken and transferred to a fresh 1.5 mL microcentrifuge tube and stored at −20 °C until required for further analysis.

### 2.4. Multilocus Sequence Typing of L. monocytogenes

Seven housekeeping loci (*abc*Z, *bgl*A, *cat*, *dap*, *dat*, *ldh*, and *lhk*A) were selected for the characterization of *L. monocytogenes* isolates by MLST. The primers that were used in this study to determine the MLST scheme are based on the panel designed by Ragon et al., 2008 and Salcedo et al., 2003 [15,34]. The following temperature conditions were used: 1 cycle 94 °C, 4 min; 35 cycles (94 °C, 30 s; 52 °C, 30 s; 72 °C, 2 min); 1 cycle 72 °C, 10 min for the PCR amplification. An annealing temperature of 52 °C was used for all genes except for *bgl*A (45 °C) (https://bigsdb.pasteur.fr/listeria, accessed on 25 March 2024).

### 2.5. Antibiogram Profiling

The antibiogram signatures of *L. monocytogenes* isolates were evaluated according to the liquid microdilution method reported by the European Committee on Antimicrobial Susceptibility Testing (EUCAST, ECOFF). Antibiotic susceptibility testing was performed using Sensititre^TM^ GPN3F plates (Thermo Scientific, Rockville, MD, USA) which are specially manufactured for Gram-positive bacteria. These specific antibiotics were selected for susceptibility testing because they are commonly used to treat infections caused by Gram-positive bacteria, including *L. monocytogenes*. These plates contained a range of 18 antibiotics at varying concentrations: erythromycin ERY (0.25–4 mg/L), clindamycin CLI (0.12–2 mg/L), gentamicin GEN (500 mg/L), streptomycin STR (1000 mg/L), quinupristin/dalfopristin SYN (0.12–4 mg/L), daptomycin DAP (0.25–8 mg/L), vancomycin VAN (1–128 mg/L), tetracycline TET (2–16 mg/L), ampicillin AMP (0.12–16 mg/L), gentamicin GEN (2–16 mg/L), rifampin RIF (0.5–4 mg/L), levofloxacin LEVO (0.25–8 mg/L), linezolid LZD (0.5–8 mg/L), penicillin PEN (0.06–8 mg/L), ciprofloxacin CIP (0.5–2 mg/L), trimethoprim/sulfamethoxazole SXT (0.5/9.5–4/76 mg/L), ceftriaxone AXO (8–64 mg/L), gatifloxacin GAT (1–8 mg/L), and oxacillin + 2% NaCl OXA+ (0.25–8 mg/L) [35,36]. The determination of the minimum inhibitory concentration (MIC) in this study followed the broth microdilution method in accordance with ISO 20776-1 [37]. First, tryptic soy agar was used as a solid medium for the cultivation of *L. monocytogenes* isolates from which colonies were standardized to the McFarland equivalent of 0.5 McF, and then verified by OD measurement using UV-1800 spectrophotometer (Shimandzu, Cole-Parmer, Durham, NC, USA). Next, 30 µL of inoculum was transferred to 11 mL of Mueller–Hinton broth (MHB, Merck, Darmstadt, Germany) and 50 µL was pipetted into microdilution panels wells which were sealed and incubated at 37 °C for 18–24 h. For the evaluation of the quality of the technique, certified strains were used, as a negative control strain, *Staphylococcus aureus* ATCC 29213 was used, while as a positive control of *L. monocytogenes* 1/2a ATCC 13932 and 4b ATCC 35152 were used. The isolate’s susceptibility was categorized as susceptible (S) and resistant (R) to each of the antimicrobials, in line with the result obtained from the susceptibility testing using standard reference documents [38].

### 2.6. Calculation and Interpretation of Multiple/Antibiotic Resistance Indices Multiple/Antibiotic Resistance Index

For each isolate of *L. monocytogenes* that showed phenotypic resistance against three or more classes of antibiotics from the panel of 18 antibiotics tested, multiple antibiotic resistance phenotypes (MARPs) were computed. The multiple antibiotic resistance index (MARI) was calculated using the method from Krumperman [39]. The calculation and interpretation of the MAR Index for each strain was conducted according to the mathematical formula MARI = a/b, where “a” represents the number of antibiotics to which an isolate was resistant, and “b” represents the total number of antibiotics tested. If MARI > 0.2, it is interpreted that those antibiotics are frequently consumed in that area and represent a high risk of antibiotic resistance. Furthermore, the antimicrobial resistance index (ARI = a/b) was evaluated for each food category, where a = the aggregate antibiotic resistance score of all isolates from a particular sample type while b = the number of antibiotics × no. of isolates from the sample type.

## 3. Results

### 3.1. Multilocus Sequence Typing

In this study, 114 strains of *L. monocytogenes* were examined molecularly for the determination of multilocus sequence types (MLST) and antimicrobial susceptibility.

Sixteen different clonal complex–sequence-type (CC–ST) allelic numbers were identified using the BIGSdb-Pasteur MLST database of *L. monocytogenes* (https://bigsdb.pasteur.fr/listeria, accessed on 25 March 2024): CC1, CC2, CC3, CC4, CC6, CC7, CC8, CC9, CC14, CC26, CC29, ST32, CC37, CC87, CC121, and CC315 (Table S1 and Table 1).

Clone CC9–ST9 had the highest percentage of isolates (29.8%), followed by clones CC8–ST8 (8.77%), CC6–ST6 (7.89%), CC29–ST29 (7.02%), and CC2–ST2 with (6.14%), followed by other clones with a percentage lower than (5.26%). Table 1 summarizes information on isolates from six years of monitoring for the presence of *L. monocytogenes*.

The multilocus sequence typing (MLST) analysis of these strains provided a comprehensive overview of their genetic diversity, identifying allelic profiles for 21 distinct sequence types (STs). These included ST2, ST3, ST4, ST6, ST7, ST8, ST9, ST12, ST14, ST26, ST29, ST32, ST37, ST87, ST121, ST145, ST328, ST399, ST520, ST580, and ST710 (Table 1 and Table S1). This diversity reflects a broad representation of genotypes across the isolates, underscoring the genetic variability within the strains analyzed. Among the multilocus sequence typing (MLST) clones isolated from meat and meat products, which constituted the largest group in this study (*n* = 80), a total of 16 unique sequence types (STs) were identified. Clones ST2, ST3, ST4, ST12, and ST710 were the least numerous with only 1 isolate or (1.25%) of the total isolates identified from meat products, followed by ST29 and ST520 with two isolates each (2.5%), with three STs or (3.75%) clones ST328 and ST399. The STs ST145 and ST580 each had four isolates (5.0%), followed by ST7 and ST37 with five isolates (6.25%) and ST8 with seven isolates (8.75%), while ST6 was identified in nine isolates (11.25%). The most represented ST in this study was ST9 from 31 (38.75%) isolates.

In dairy products, 11 STs were identified from 25 isolates each with one (4.0%) ST14, ST37, ST87, and ST520 identified, followed by ST4, ST8, ST9, ST26, and ST32 with two each (8.0%). ST145 was identified in five (20.0%) isolates, and ST29 was identified in six (24.0%) isolates. Fish meat products were represented by one (25.0%) of four STs: ST2, ST26, ST87, and ST145, and also by one (25.0%) of four isolates of combined food products identified: ST6, ST8, ST9, and ST121. The only isolate from environmental samples was identified as ST7.

### 3.2. Antimicrobial Susceptibility

Of all isolates tested (Table 2 and Table S1) to the panel of 18 antibiotics at different concentrations, resistance against the largest number of antibiotics was observed in isolates from meat products 13/18 (72.2%), while 5/18 (27.8%) were susceptible. Isolates from dairy products showed resistance against 5/18 (27.8%) of the antimicrobials tested, while 13/18 (72.2%) were susceptible to the antibiotics tested. Isolates from fish products appeared resistant against 11/18 (61.1%) antimicrobials whereas *L. monocytogenes* were susceptible to 7/18 (38.9%). Isolates from the environmental sample showed resistance against 3/18 (16.7%) of the antibiotics tested while 15/18 (83.3%) were susceptible to the antibiotics. Of the combined food products, resistance against 6/18 (33.3%) antibiotics was observed while the isolates were susceptible to 12/18 (66.7%) of antimicrobials.

**Figure 1:**
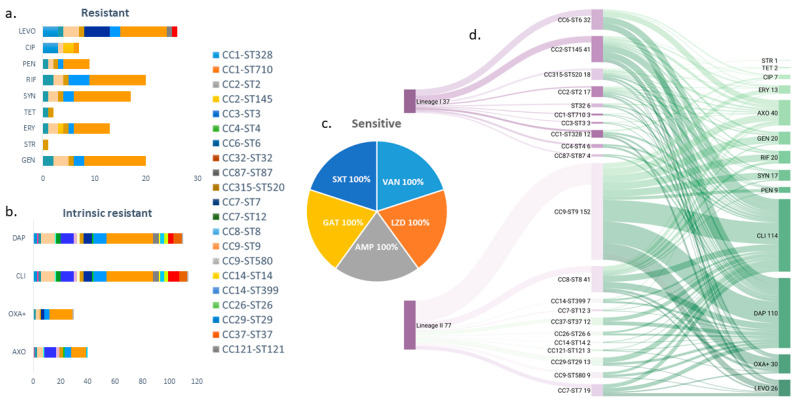
Overview of *L. monocytogenes* strains (sequence type–ST) isolated from the food chain in Kosovo. (**a**) Strains resistant against nine emergency antimicrobials LEVO, CIP, PEN, RIF, SYN, TET, ERY, STR, and GEN, shown in respective colors for each CC-STs. (**b**) Four antibiotics, which *L. monocytogenes*, are naturally resistant (intrinsic resistance) to DAP, CLI, OXA+, and AXO, shown in respective colors for each CC-STs. (**c**) A pie chart displays that all isolates are sensitive to VAN, SXT, LZD, GAT, and AMP. (**d**) Sankey chart—correlation of antibiotics to CC–ST and phylogenetic lineage I and II.

The susceptibility of *L. monocytogenes* isolates to panels of antimicrobials that were evaluated in this study varied differently (Figure 1). To daptomycin, all isolates were resistant 114/114 (100%); to clindamycin 110/114 (96.5%); ceftriaxone 40/114 (35.1%); oxacillin + 2% NaCl 30/114 (26.3%); levofloxacin 26/114 (22.8%); gentamicin and rifampin 20/114 (17.5%); quinupristin/dalfopristin 17/114 (14.9%); erythromycin 13/114 (11.4%); penicillin 9/114 (7.89%); ciprofloxacin 7/114 (6.14%); tetracycline 2/114 (1.75%); while 1/114 (0.88%) showed resistance against streptomycin. However, the isolates were susceptible to antimicrobials including ampicillin, gatifloxacin, vancomycin, linezolid, and trimethoprim/sulfamethoxazole.

The highest multi-resistant clones against antimicrobials were CC9–ST9 at (16.7%), followed by CC6–ST6 at (8.77%). CC2–ST145 and CC8–ST8 were (6.14%) each. CC7–ST7 (4.39%), CC1–ST328 (2.63%), ST32 and CC315–ST520 were (1.75%) each, as well as CC1–ST710, CC3–ST3, CC7–ST12, CC9–ST580, CC14–ST399, CC29–ST29 and CC121–ST121 at each (0.88%). Among the clones that did not show multi-resistance to antimicrobials were CC4–ST4, CC14–ST14, CC26–ST26, CC37–ST37, and CC87–ST87 (Table S1 and Figure 1).

### 3.3. Minimum Inhibitory Concentration

Table 3 summarizes the antimicrobial resistance profiles with details on the minimum inhibitory concentration (MIC) values of *L. monocytogenes* isolates against a panel of 18 antibiotics. The resistance and MIC values were interpreted according to the EUCAST document [38]. These data provide insight into resistance patterns, highlighting which antibiotics are less effective against the isolates and offering a valuable reference for understanding the potential challenges in treating infections caused by these strains.

Thick line breakpoints were used for interpretation of the antimicrobial susceptibility of *L. monocytogenes* based on the EUCAST (erythromycin, penicillin, ampicillin and trimethoprim/sulfamethoxazole); breakpoints for *Staphylococcus aureus* were adopted for the following antibiotics: gentamicin, tetracycline, quinupristin/dalfopristin, rifampin, ciprofloxacin, levofloxacin, clindamycin, daptomycin, vancomycin, linezolid, and gatifloxacin; intrinsic resistance (INT) antibiotics ceftriaxone, oxacillin + 2% NaCl, and clindamycin; streptomycin with a concentration of 1000 mg/L; and (S) Sensitive and (R) Resistant. The grey zone represents the concentration range for each antimicrobial from the GPN3F plate.

### 3.4. Multiple Antibiotic Resistance

The antibiotic resistance patterns and MAR index are presented in Table 4. Isolates from the samples exhibited 27 resistance patterns against 18 antimicrobials ranging from 3 to 12 antibiotics. The CLI–DAP and CLI–DAP–AXO phenotypes (*n* = 45 and *n* = 19, respectively) were the most occurring phenotypes observed. All of the isolates showed resistance against at least one of the antibiotics tested: 4 (3.5%) isolates showed resistance against one antibiotic, 45 (39.5%) were resistant against 2 antibiotics, 32 (28.1%) showed resistance against three antibiotics, while 65 (57.0%) showed multidrug-resistant phenotypes against the antibiotics tested. The antibiotic resistance index (ARI) of each isolate ranged from 0.06 to 0.67 (Table S1). Out of all strains tested, 34/114 (29.8%) had MAR values greater than >0.2. The ARI of each of the samples in each category including milk and milk products (MMP, 0.22) and fish meat products (FMP, 0.26) were >0.2 of the permissible Krumperman threshold while the environmental sample (ES, 0.05), meat and meat products (MP, 0.12), and combined food products (CFP, 0.18) fall within the threshold.

## 4. Discussion

Listeriosis and its treatment with adequate antibiotics is one of the current challenges not only in Europe but also in Kosovo. The excessive and uncontrolled administration of antibiotics in animals contributes to antimicrobial resistance and this presents further risks related to the treatment of listeriosis, increasing the severity of the disease and treatment costs, reducing the effect of antibiotics, and spreading resistant genes. Given that food products are the main source of listeriosis infections, such a study in our country is at the service of implementing effective measures in food safety through continuous surveillance for resistance, and this could lead to the mitigation of health risks caused by *L. monocytogenes*. Although there have been some studies regarding the prevalence of *L. monocytogenes* from foods and the characterization of isolates [19], data regarding the antimicrobial resistance are limited in Kosovo and so far have been described in isolates from sporadic ovine listeria meningoencephalitis cases [40]. However, information is available on the prevalence, antimicrobial resistance, and the molecular characteristics of *L. monocytogenes* isolates from the food chain worldwide [21,23,41,42,43,44,45,46,47,48].

The identification of clonal complexes and sequence types, as well as the MIC testing against a panel of 18 antibiotics of 114 strains of *L. monocytogenes* isolated from food products from 2016 to 2022, provided representative data of isolates and antimicrobial resistant *L. monocytogenes* circulating in Kosovo. The same results as those obtained by GenoListeria [49], where sixteen groups of clonal complexes (CCs) of *L. monocytogenes* from the food chain in Kosovo were identified, were also obtained by the MLST determination method [19].

From all isolates of *L. monocytogenes* grouped into 16 clonal complexes in our study by MLST, 21 different ST groups were identified. Similar CC–STs were found among isolates in studies carried out in many countries around the world in the food chain. Thus, CC14–ST399, CC7–ST7, CC8–ST8, and CC1–ST328, were isolated from Danish ready-to-eat food samples [50]. In China, the study conducted from 2010 to 2019 highlights that in meat products the most predominant isolates belonged to CC9–ST9 clones [51], while the most frequent strains isolated in Poland from meat processing plant environments were CC2–ST2, CC2–ST145, CC3–ST3, CC6–ST6 and CC87–ST87 [52]. In another study carried out on foodstuffs, the environment and clinical samples in Italy, CC14–ST14, CC26–ST26, CC29–ST29, ST32, CC37–ST37 and CC315–ST520 were reported [23]. In Poland, the most frequent isolates recovered from food products were CC9–ST9, CC121–ST121 and CC9–ST580 [53], while CC7–ST12 and CC1–ST328 were among the most frequent isolates recovered from the food production chain in Australia [54]. However, CC1–ST710, which belongs to Lineage I and Serogroup IVb, was not isolated from food products except from clinical cases from a study conducted in northern Taiwan [55]. Similar findings were reported by Daza Prieto B. et al. who characterized 160 isolates of *L. monocytogenes* obtained from different food sources in Montenegro from 2014 to 2022. Similar distributions of ST8, ST9, and ST121 were observed except for ST 155 [56].

In our study, the clone CC9–ST9 is the most represented, which originates mainly from meat products with a prevalence of 27.2%. A similar result was found in studies carried out on 300 genotypes from five continents [57]. Another study in France reported CC9 as the most frequently observed isolate recovered from meat products [58]. Furthermore, ST9 was also reported as the most widespread strain observed in a study on meat products in Norway [59].

Our study showed that *L. monocytogenes* showed the highest antimicrobial resistant against clindamycin (100%) and daptomycin, while it was lower for tetracycline and streptomycin and no resistance was observed against vancomycin, ampicillin, linezolid, gatifloxacin, and trimethoprim–sulfamethoxazole. Similar data in the previous studies performed earlier in Pacific Northwest, Canada, Jordan, and Asia have been reported [21,42]. The antibiogram profile of *L. monocytogenes* isolates in our study showed high sensitivity (100%) to vancomycin, ampicillin, linezolid, gatifloxacin, and trimethoprim–sulfamethoxazole. A similar sensitivity of *L. monocytogenes* isolates to these antibiotics except gatifloxacin was observed in a study of food production factories in Germany [46]. High resistance has also been reported against clindamycin (100%) [41,42], daptomycin (96.5%) [46], amoxicillin (35.1%), [43] and oxacillin+ 2% NaCl (24.6%) [23]. In many previous studies, lower resistance of *L. monocytogenes* isolates levofloxacin (22.8%) [47], gentamicin, and rifampicin [45] from (17.5%), to quinupristin/dalfopristin (14.9%) [23], erythromycin (11.4%) [44], penicillin (7.89%), ciprofloxacin (6.14%), and tetracycline (1.75%) [47] have been reported. Otherwise, in the conducted studies, streptomycin was sensitive [44,47], while in our study, one isolate (0.88%) was resistant to streptomycin.

The MARI indices indicate the number of phenotypic resistances observed against the tested antimicrobial agents and vary among the isolates. A high MARI score for an isolate suggests it exhibits resistance against multiple antibiotics, highlighting its significant level of resistance. The ARI of the food category including meat and meat products and fish meat products were >0.2 of the permissible Krumperman threshold. A study carried out in ready-to-eat products including meat and fish products also exceeded the ARI threshold of >0.2 and a similarity with the values of our study is observed [43].

This could constitute a potential threat to food safety and public health in the Kosovo region. In addition, 57.0% of the isolates displayed multidrug-resistant (MDR) phenotypes against the tested antibiotics. Other authors have noted the varying prevalence of MDR, in North Africa (41.86%), in Spain (54.0%), and in Turkey (73.91%) [41,60,61]. MDR bacteria are particularly challenging to treat, often leading to higher hospitalization costs and extended antibiotic treatments. These MDR strains are frequently found in the environment and can infiltrate food processing plants through factory personnel or raw materials. Various factors contribute to antimicrobial resistance in *L. monocytogenes*, especially in the food sector. Additionally, stress from food processing can promote the development of resistance to clinically relevant antibiotics. To conclude, this study revealed the profiles of antimicrobial resistance in correlation with the food category and the sequence types of *L. monocytogenes* strains identified and highlighted the possible risks that could arise from the consumption of these foods. It is worth noting that high resistance against clindamycin, ceftriaxone, oxacillin + 2% NaCl, levofloxacin, gentamicin, rifampin, quinupristin/dalfopristin, erythromycin, penicillin, ciprofloxacin, tetracycline, and streptomycin were observed in this study. Compared to previous studies in different countries around the world, our results suggest differing important trends in *L. monocytogenes* resistance against antimicrobials isolated from food. These results also showed that the CC9–ST9 isolates, which are the most represented, showed over 66% resistance to the panel of antibiotics used in this study. However, in the current study, we did not address changes in antimicrobial patterns over time during this period due to the uneven distribution of positive samples per year. Therefore, the evolution of antimicrobial resistance remains to be assessed on an annual basis in the future.

The current study describes the antimicrobial resistance of *L. monocytogenes* against the tested panel of 18 antimicrobials. However, we did not address changes in antimicrobial patterns over time during this period. Therefore, evolution of antimicrobial resistance must be assessed on an annual basis.

## 5. Conclusions

This study analyzed 114 *L. monocytogenes* isolates recovered from the food chain over six years, classified into 16 clonal complexes and 21 sequence types. The susceptibility of these isolates to a panel of 18 antimicrobials was evaluated, highlighting critical implications for food safety. A concerning trend of increased resistance was observed, particularly for levofloxacin, gentamicin, and rifampicin, aligning with broader regional and global reports of rising antimicrobial resistance (AMR) in *L. monocytogenes*. Multidrug-resistance (MDR) was especially notable in isolates from meat products, with resistance to more than three antibiotic classes. These findings revealed that food products of animal origin can be a potential reservoir for the transmission of antimicrobial resistant *L. monocytogenes* signalling the need for targeted surveillance and control measures. This study’s results advocate for the continuous monitoring of *L. monocytogenes* epidemiological clones as well as AMR monitoring throughout the food chain in Kosovo to enhance public health and safety.

## Figures and Tables

**Table 1:** Genetic characteristics of *L. monocytogenes* isolates originating from food products.

Molecular Genotype of Isolates				Strain Origin (No. of Isolates)				
Lineage	Serotype Group	Clonal Complex	Sequence Type	MP	MDP	FMP	ES	CFP
I	IVb	CC1	ST328	3				
			ST710	1				
	IVb	CC2	ST2	1	5	1		
			ST145	4		1		
	IIb	CC3	ST3	1				
	IVb	CC4	ST4	1	2			
	IVb	CC6	ST6	9				1
	IVb	ST32	ST32		2			
	IIb	CC87	ST87		1	1		
	IVb	CC315	ST520	2	1			
II	IIa	CC7	ST7	5			1	
			ST12	1				
	IIa	CC8	ST8	7	2			1
	IIc	CC9	ST9	31	2			1
			ST580	4				
	IIa	CC14	ST14		1			
			ST399	3				
	IIa	CC26	ST26		2	1		
	IIa	CC29	ST29	2	6			
	IIa	CC37	ST37	5	1			
	IIa	CC121	ST121					1

MP—Meat and meat products; MDP—Milk and dairy products; FMP—Fish meat products; ES—Environment sample; CFP—Combined food products.

**Table 2:** Antimicrobial susceptibility of *L. monocytogenes* distributed across phylogenetic lineages and food categories.

Phylogenetic Lineage	Food Category	Resistant	Intrinsic Resistance	Sensitive
Lineage I (*n* = 37)	MP (*n* = 22)	GEN (5), STR (1), ERY (4), TET (1), SYN (3), RIF (4), CIP (6), PEN (1), LEVO (6)	AXO (15), OXA+ (4), CLI (22), DAP (22)	VAN, AMP, LZD, GAT, SXT all by (22)
	MMP (*n* = 11)	PEN (1), LEVO (1)	AXO (4), CLI (11), DAP (11)	VAN, AMP, LZD, GAT, SXT all by (11)
	FMP (*n* = 3)	GEN (1), ERY (1), TET (1), SYN (1), RIF (1), PEN (1), LEVO (1)	AXO (2), OXA+ (2), CLI (3), DAP (3)	VAN, AMP, LZD, GAT, SXT all by (3)
	CFP (*n* = 1)	-	AXO (1), CLI (1), DAP (1)	VAN, AMP, LZD, GAT, SXT all by (1)
Lineage II (*n* = 77)	MP (*n* = 58)	GEN (13), ERY (8), SYN (13), RIF (14), CIP (1), PEN (6), LEVO (16)	AXO (18), OXA+ (22), CLI (58), DAP (56)	VAN, AMP, LZD, GAT, SXT all by (58)
	MMP (*n* = 14)	LEVO (1)	CLI (14), DAP (12)	VAN, AMP, LZD, GAT, SXT all by (14)
	FMP (*n* = 1)	-	CLI (1), DAP (1)	VAN, AMP, LZD, GAT, SXT all by (1)
	ES (*n* = 1)	LEVO (1)	CLI (1), DAP (1)	VAN, AMP, LZD, GAT, SXT all by (1)
	CFP (*n* = 3)	GEN (1), RIF (1)	OXA+ (2), CLI (3), DAP (3)	VAN, AMP, LZD, GAT, SXT all by (3)

MP—Meat and meat products; MMP—Milk and milk products; FMP—Fish meat products; ES—Environment sample; CFP—Combined food products.

**Table 3:** The minimum inhibitory concentration (MIC) distribution of the 114 *L. monocytogenes* strains.

Antimicrobials	MIC (mg/L)													Interpretation	
	0.06	0.12	0.25	0.50	1	2	4	8	16	32	64	128	1000	S	R
Gentamicin						2	1	3	16					≤2	>2
Streptomycin													1	<1000	≥1000
Erythromycin			31	6	3	9	4							≤1	>1
Tetracycline						1	0	0	1					≤1	>1
Quinupristin/ Dalfopristin		5	36	54	2	0	17							≤1	>1
Rifampin	0	0	0	3	2	1	14							≤0.06	>0.06
Ciprofloxacin				81	24	7								≤0.001	>2
Penicillin	7	50	26	2	9	9	0	0						≤1	>1
Levofloxacin			0	88	25	0	1							≤0.001	>1
Ceftriaxone								32	24	9	31			<32	≥32
Oxacillin + 2% NaCl			4	7	33	40	3	27						<2	≥4
Clindamycin		0	11	16	51	36								<0.25	≥0.25
Daptomycin			0	0	4	29	62	19						≤1	>1
Vancomycin					7	0	0	0	0	0	0	0		≤2	>2
Ampicillin		51	15	8	1	0	0	0	0					≤1	>1
Linezolid				9	62	42	0	0						≤4	>4
Gatifloxacin					0	0	0	0						≤1	>1
Trimethoprim/Sulfamethoxazole				0	0	0	0							≤4/76	>4/76

**Table 4:** Multiple antibiotic resistance phenotypes of *L. monocytogenes* (MARPs) strains.

S/N	Antibiotic Resistance Phenotypes MARPs Patterns	No. of Antibiotics	MARI	Freq
1	CLI–DAP–AXO	3	0.17	19
2	LEVO–CLI–DAP	3	0.17	7
3	OXA+–CLI–DAP	3	0.17	4
4	GEN–CLI–DAP	3	0.17	1
5	CIP–CLI–DAP	3	0.17	1
6	OXA+–LEVO–CLI–DAP	4	0.22	4
7	LEVO–CIP–CLI–DAP	4	0.22	3
8	CIP–CLI–DAP–AXO	4	0.22	1
9	ERY–CLI–DAP–AXO	4	0.22	1
10	OXA+–CLI–DAP–RIF	4	0.22	1
11	OXA+–CIP–CLI–DAP	4	0.22	1
12	OXA+–CLI–DAP–AXO	4	0.22	1
13	PEN–LEVO–CLI–DAP	4	0.22	1
14	OXA+–LEVO–CLI–DAP–RIF	5	0.28	1
15	GEN–CLI–SYN–DAP–AXO–RIF	6	0.33	1
16	OXA+–GEN–CLI–DAP–AXO–RIF	6	0.33	1
17	OXA+–GEN–CLI–SYN–DAP–AXO–RIF	7	0.39	1
18	OXA+–LEVO–CIP–GEN–ERY–CLI–DAP	7	0.39	1
19	OXA+–LEVO–GEN–CLI–SYN–DAP–AXO–RIF	8	0.44	2
20	OXA+–GEN–ERY–CLI–SYN–DAP–AXO–RIF	8	0.44	2
21	PEN–OXA+–GEN–CLI–SYN–DAP–AXO–RIF	8	0.44	1
22	PEN–OXA+–GEN–ERY–CLI–SYN–DAP–AXO–RIF	9	0.50	3
23	OXA+–LEVO–GEN–ERY–CLI–SYN–DAP–AXO–RIF	9	0.50	3
24	PEN–OXA+–LEVO–GEN–CLI–SYN–DAP–AXO–RIF	9	0.50	1
25	PEN–OXA+–LEVO–GEN–ERY–CLI–SYN–DAP–AXO–RIF	10	0.56	1
26	PEN–OXA+–LEVO–GEN–ERY–CLI–SYN–DAP–TET–AXO–RIF	11	0.61	1
27	PEN–OXA+–LEVO–GEN–STR–ERY–CLI–SYN–DAP–TET–AXO–RIF	12	0.67	1

GEN—gentamicin; STR—streptomycin; ERY—erythromycin; TET—tetracycline; SYN—quinupristin/dalfopristin; RIF—rifampin; PEN—penicillin; CIP—ciprofloxacin; LEVO—levofloxacin; OXA+—oxacillin + 2% NaCl; AXO—ceftriaxone; CLI—clindamycin; DAP—daptomycin.

## Data Availability

The original contributions presented in the study are included in the article/Supplementary Material, further inquiries can be directed to the corresponding author.

## Supplementary Materials

The following supporting information can be downloaded at: https://www.mdpi.com/article/10.3390/microorganisms12122441/s1, Table S1: Genetic profiles of antibiotic resistant of *L. monocytogenes* isolates and antimicrobial resistance phenotypes/indices of *L. monocytogenes* strains from food samples.
