# Supplementary material for: Multilocus Sequence Typing and Antimicrobial Susceptibility of Listeria monocytogenes Isolated from Foods Surveyed in Kosovo
Source: Microorganisms. 2024 Nov 27;12(12):2441. doi: 10.3390/microorganisms12122441 (PMC11677359; doi:10.3390/microorganisms12122441)
Supplement: Supplementary file 1 [file microorganisms-12-02441-s001.zip › microorganisms-3270115-supplementary.pdf]

Table S1. Genetic profiles of antibiotic resistant of *L. monocytogenes* isolates and antimicrobial resistance phenotypes/indices of *L. monocytogenes* strains from food samples

S/N	Food types	Year	Lineages	CC-STs	Antibiotic resistance phenotypes (M)ARPs patterns	No. of antibiotics	MARI
1	MP	2016	I	ST32	CLI-DAP-AXO	3	0.17
2	MP	2016	II	CC14-ST14	CLI-DAP	2	0.11
3	MP	2018	II	CC26-ST26	CLI-DAP	2	0.11
4	MP	2018	II	CC8-ST8	CLI-DAP	2	0.11
5	MP	2018	I	CC2-ST145	CLI-DAP	2	0.11
6	MP	2018	I	CC87-ST87	CLI-DAP	2	0.11
7	MP	2018	II	CC29-ST29	CLI	1	0.06
8	MP	2018	I	CC2-ST145	PEN-LEVO-CLI-DAP	4	0.22
9	MP	2018	I	CC2-ST145	CLI-DAP	2	0.11
10	MP	2018	I	CC2-ST145	CLI-DAP	2	0.11
11	MP	2018	II	CC8-ST8	CLI-DAP	2	0.11
12	MP	2019	II	CC9-ST9	CLI-DAP	2	0.11
13	MP	2019	I	ST32	CLI-DAP-AXO	3	0.17
14	MP	2019	II	CC9-ST9	CLI-DAP	2	0.11
15	MP	2019	I	CC315-ST520	CLI-DAP-AXO	3	0.17
16	MP	2019	I	CC2-ST145	CLI-DAP-AXO	3	0.17
17	MP	2019	II	CC26-ST26	CLI-DAP	2	0.11
18	MP	2021	II	CC37-ST37	CLI-DAP	2	0.11
19	MP	2021	II	CC29-ST29	CLI-DAP	2	0.11
20	MP	2021	I	CC4-ST4	CLI-DAP	2	0.11
21	MP	2021	I	CC4-ST4	CLI-DAP	2	0.11
22	MP	2021	II	CC29-ST29	CLI-DAP	2	0.11
23	MP	2021	II	CC29-ST29	CLI-DAP	2	0.11
24	MP	2021	II	CC29-ST29	CLI	1	0.06
25	MP	2021	II	CC29-ST29	LEVO-CLI-DAP	3	0.17
ARI							0.12
1	MMP	2016	II	CC9-ST580	CLI-DAP	2	0.11
2	MMP	2016	II	CC9-ST580	CLI-DAP	2	0.11
3	MMP	2016	II	CC9-ST9	CLI-DAP	2	0.11
4	MMP	2016	II	CC14-ST399	CLI-DAP	2	0.11
5	MMP	2016	I	CC6-ST6	CIP-CLI-DAP	3	0.17
6	MMP	2016	II	CC8-ST8	OXA+-LEVO-GEN-CLI-SYN-DAP-AXO-RIF	8	0.44
7	MMP	2016	I	CC6-ST6	CLI-DAP-AXO	3	0.17
8	MMP	2016	II	CC8-ST8	CLI-DAP-AXO	3	0.17
9	MMP	2016	II	CC14-ST399	CLI-DAP	2	0.11
10	MMP	2016	II	CC8-ST8	CLI-DAP-AXO	3	0.17
11	MMP	2016	I	CC6-ST6	CLI-DAP-AXO	3	0.17
12	MMP	2016	II	CC14-ST399	CLI-DAP-AXO	3	0.17
13	MMP	2017	II	CC9-ST9	CLI-DAP	2	0.11
14	MMP	2017	I	CC6-ST6	CLI-DAP-AXO	3	0.17
15	MMP	2017	I	CC6-ST6	CIP-CLI-DAP-AXO	4	0.22
16	MMP	2017	I	CC6-ST6	CLI-DAP-AXO	3	0.17
17	MMP	2017	II	CC37-ST37	CLI-DAP	2	0.11
18	MMP	2017	I	CC6-ST6	ERY-CLI-DAP-AXO	4	0.22
19	MMP	2017	I	CC6-ST6	CLI-DAP-AXO	3	0.17
20	MMP	2017	II	CC37-ST37	CLI-DAP	2	0.11
21	MMP	2017	II	CC9-ST9	CLI-DAP	2	0.11
22	MMP	2017	I	CC6-ST6	CLI-DAP-AXO	3	0.17
23	MMP	2017	II	CC37-ST37	CLI-DAP	2	0.11
24	MMP	2018	II	CC9-ST9	CLI-DAP	2	0.11
25	MMP	2018	II	CC9-ST9	CLI-DAP	2	0.11
26	MMP	2018	II	CC9-ST9	PEN-OXA+-GEN-ERY-CLI-SYN-DAP-AXO-RIF	9	0.5
27	MMP	2018	II	CC29-ST29	CLI	1	0.06
28	MMP	2018	II	CC9-ST9	CLI-DAP	2	0.11
29	MMP	2018	II	CC8-ST8	CLI-DAP	2	0.11
30	MMP	2018	II	CC7-ST7	CLI-DAP	2	0.11
31	MMP	2018	II	CC37-ST37	CLI-DAP	2	0.11
32	MMP	2018	II	CC9-ST9	CLI-DAP	2	0.11
33	MMP	2019	I	CC315-ST520	PEN-OXA+-LEVO-GEN-STR-ERY-CLI-SYN-DAP-TET-AXO-RIF	12	0.67
34	MMP	2019	II	CC9-ST9	CLI-DAP	2	0.11
35	MMP	2019	II	CC9-ST9	CLI-DAP	2	0.11
36	MMP	2019	II	CC7-ST7	LEVO-CLI-DAP	3	0.17
37	MMP	2019	II	CC9-ST580	LEVO-CLI-DAP	3	0.17
38	MMP	2019	II	CC9-ST9	CLI-DAP	2	0.11
39	MMP	2019	II	CC9-ST9	OXA+-GEN-ERY-CLI-SYN-DAP-AXO-RIF	8	0.44
40	MMP	2019	II	CC9-ST9	CLI-DAP	2	0.11
41	MMP	2019	II	CC9-ST9	LEVO-CLI-DAP	3	0.17
42	MMP	2019	I	CC1-ST328	LEVO-CIP-CLI-DAP	4	0.22
43	MMP	2019	II	CC9-ST9	CLI-DAP	2	0.11
44	MMP	2019	II	CC8-ST8	CLI-DAP-AXO	3	0.17
45	MMP	2019	II	CC9-ST9	OXA+-LEVO-GEN-ERY-CLI-SYN-DAP-AXO-RIF	9	0.5
46	MMP	2019	II	CC9-ST9	PEN-OXA+-GEN-CLI-SYN-DAP-AXO-RIF	8	0.44
47	MMP	2019	II	CC9-ST9	PEN-OXA+-LEVO-GEN-CLI-SYN-DAP-AXO-RIF	9	0.5
48	MMP	2019	II	CC9-ST9	PEN-OXA+-GEN-ERY-CLI-SYN-DAP-AXO-RIF	9	0.5
49	MMP	2019	II	CC9-ST9	PEN-OXA+-LEVO-GEN-ERY-CLI-SYN-DAP-AXO-RIF	10	0.56
50	MMP	2019	II	CC8-ST8	PEN-OXA+-GEN-ERY-CLI-SYN-DAP-AXO-RIF	9	0.5
51	MMP	2019	II	CC8-ST8	OXA+-LEVO-CLI-DAP-RIF	5	0.28
52	MMP	2019	II	CC9-ST9	OXA+-LEVO-GEN-ERY-CLI-SYN-DAP-AXO-RIF	9	0.5
53	MMP	2019	II	CC9-ST9	OXA+-LEVO-CLI-DAP	4	0.22
54	MMP	2019	II	CC9-ST9	OXA+-CIP-CLI-DAP	4	0.22
55	MMP	2019	II	CC9-ST9	OXA+-LEVO-CLI-DAP	4	0.22
56	MMP	2019	II	CC9-ST9	OXA+-LEVO-GEN-CLI-SYN-DAP-AXO-RIF	8	0.44
57	MMP	2019	II	CC9-ST9	OXA+-LEVO-GEN-ERY-CLI-SYN-DAP-AXO-RIF	9	0.5
58	MMP	2019	II	CC9-ST9	OXA+-CLI-DAP	3	0.17
59	MMP	2019	I	CC2-ST2	OXA+-GEN-CLI-DAP-AXO-RIF	6	0.33
60	MMP	2019	II	CC9-ST9	OXA+-GEN-CLI-SYN-DAP-AXO-RIF	7	0.39

61	MMP	2019	II	CC9-ST9	OXA+-CLI-DAP	3	0.17
62	MMP	2019	I	CC2-ST145	OXA+-LEVO-CIP-GEN-ERY-CLI-DAP	7	0.39
63	MMP	2019	I	CC2-ST145	OXA+-GEN-ERY-CLI-SYN-DAP-AXO-RIF	8	0.44
64	MMP	2019	I	CC2-ST145	GEN-CLI-SYN-DAP-AXO-RIF	6	0.33
65	MMP	2020	II	CC9-ST9	OXA+-CLI-DAP	3	0.17
66	MMP	2020	II	CC7-ST7	OXA+-LEVO-CLI-DAP	4	0.22
67	MMP	2021	II	CC7-ST7	OXA+-LEVO-CLI-DAP	4	0.22
68	MMP	2021	II	CC29-ST29	CLI	1	0.06
69	MMP	2021	II	CC37-ST37	CLI-DAP	2	0.11
70	MMP	2021	I	CC4-ST4	CLI-DAP	2	0.11
71	MMP	2021	II	CC9-ST580	CLI-DAP	2	0.11
72	MMP	2021	II	CC9-ST9	CLI-DAP	2	0.11
73	MMP	2021	I	CC1-ST328	LEVO-CIP-CLI-DAP	4	0.22
74	MMP	2021	I	CC1-ST328	LEVO-CIP-CLI-DAP	4	0.22
75	MMP	2021	I	CC315-ST520	CLI-DAP-AXO	3	0.17
76	MMP	2022	I	CC1-ST710	CLI-DAP-AXO	3	0.17
77	MMP	2022	II	CC7-ST7	LEVO-CLI-DAP	3	0.17
78	MMP	2022	I	CC3-ST3	CLI-DAP-AXO	3	0.17
79	MMP	2022	II	CC7-ST12	CLI-DAP-AXO	3	0.17
80	MMP	2022	I	CC2-ST145	LEVO-CLI-DAP	3	0.17
ARI							0.22
1	FMP	2018	I	CC87-ST87	CLI-DAP	2	0.11
2	FMP	2018	II	CC26-ST26	CLI-DAP	2	0.11
3	FMP	2019	I	CC2-ST145	OXA+-CLI-DAP-AXO	4	0.22
4	FMP	2020	I	CC2-ST2	PEN-OXA+-LEVO-GEN-ERY-CLI-SYN-DAP-TET-AXO-RIF	11	0.61
ARI							0.26
1	CFP	2019	II	CC6-ST6	CLI-DAP-AXO	3	0.17
2	CFP	2019	II	CC9-ST9	GEN-CLI-DAP	3	0.17
3	CFP	2019	II	CC121-ST121	OXA+-CLI-DAP	3	0.17
4	CFP	2019	I	CC8-ST8	OXA+-CLI-DAP-RIF	4	0.22
ARI							0.18
1	ES	2021	II	CC7-ST7	LEVO-CLI-DAP	3	0.17
ARI							0.05

Abbreviations: CC-ST – clonal complex-sequence type; (M)ARP – Multi antibiotic resistance phenotypes patterns; MARI – Multiple antibiotic resistance index; ARI- Antibiotic resistance index; MP – Meat products; MMP – Milk and milk products, FMP – Fish meat products; CFP – Combined food products; ES – Environmetal sample; GEN – gentamicin; STR – streptomycin; ERY – erythromycin; TET – tetracycline; SYN - quinupristin/dalfopristin; RIF – rifampin; PEN – penicillin; CIP – ciprofloxacin; LEVO – levofloxacin; OXA+ - oxacillin + 2% NaCl; AXO – ceftriaxone; CLI - clindamycin; DAP – daptomycin.
